# CD47 Promotes Protective Innate and Adaptive Immunity in a Mouse Model of Disseminated Candidiasis

**DOI:** 10.1371/journal.pone.0128220

**Published:** 2015-05-26

**Authors:** Dhammika H. M. L. P. Navarathna, Erica V. Stein, Elizabeth C. Lessey-Morillon, Debasis Nayak, Gema Martin-Manso, David D. Roberts

**Affiliations:** 1 Laboratory of Pathology, Center for Cancer Research, National Cancer Institute, Bethesda, MD 20892, United States of America; 2 National Institute of Neurological Disorders and Stroke, National Institutes of Health, Bethesda, MD 20892, United States of America; 3 Microbiology and Immunology Program of the Institute for Biomedical Sciences, Departments of Microbiology, Immunology and Tropical Medicine, George Washington University, Washington, D.C. 20037, United States of America; King's College London Dental Institute, UNITED KINGDOM

## Abstract

CD47 is a widely expressed receptor that regulates immunity by engaging its counter-receptor SIRPα on phagocytes and its secreted ligand thrombospondin-1. Mice lacking CD47 can exhibit enhanced or impaired host responses to bacterial pathogens, but its role in fungal immunity has not been examined. *cd47* ^-/-^ mice on a C57BL/6 background showed significantly increased morbidity and mortality following *Candida albicans* infection when compared with wild-type mice. Despite normal fungal colonization at earlier times, *cd47* ^-/-^ mice at four days post-infection had increased colonization of brain and kidneys accompanied by stronger inflammatory reactions. Neutrophil and macrophage numbers were significantly elevated in kidneys and neutrophils in the brains of infected *cd47* ^-/-^ mice. However, no defect in phagocytic activity towards *C*. *albicans* was observed in *cd47* ^-/-^ bone-marrow-derived macrophages, and neutrophil and macrophage killing of *C*. *albicans* was not impaired. CD47-deficiency did not alter the early humoral immune response to *C*. *albicans*. Th1, Th2, and Th17 population of CD4^+^ T cells were expanded in the spleen, and gene expression profiles of spleen and kidney showed stronger pro-inflammatory signaling in infected *cd47* ^-/-^ mice. The chemoattractant chemokines MIP-2α and MIP-2β were highly expressed in infected spleens of *cd47* ^-/-^ mice. G-CSF, GM-CSF, and the inflammasome component NLRP3 were more highly expressed in infected *cd47* ^-/-^ kidneys than in infected wild-type controls. Circulating pro- (TNF-α, IL-6) and anti-inflammatory cytokines (IL-10) were significantly elevated, but IL-17 was decreased. These data indicate that CD47 plays protective roles against disseminated candidiasis and alters pro-inflammatory and immunosuppressive pathways known to regulate innate and T cell immunity.

## Introduction

*Candida albicans* is a commensal in the gastrointestinal tract and the most common human fungal pathogen [1]. It is the fourth leading cause of nosocomial infections in the United States [2]. Disseminated candidiasis is often fatal in immunocompromised individuals, but superficial oral thrush and vaginal yeast infections can also affect immunocompetent individuals [3]. Virulence attributes of *C*. *albicans* include secretion of extracellular proteases and carbon monoxide, expression of host adhesion receptors, and phenotypic switching to invasive hyphal forms during infection [4,5]. These virulence attributes enable invasion of host tissues and also control the host immune response by altering immune cell differentiation and cytokine production [4]. A better understanding of this host-pathogen cross-talk during disseminated candidiasis is needed to identify targets for novel drugs to supplant existing antifungal agents and counter emerging drug resistance.

Several aspects of human disseminated candidiasis can be effectively modeled in mice and have contributed to our present understanding of cell mediated and innate immune responses to *C*. *albicans* infection [6]. Neutrophils are the primary defense against a *C*. *albicans* infection, whereas macrophages coordinate cell-mediated and innate immunity. *C*. *albicans* infections alter the Th1/Th2/Th17 immune balance and systemic cytokine and chemokine expression. Virulent candidemia induces Th2 subset activation, thereby suppressing the Th1 subset and increasing susceptibility to infection [7,8]. Conversely, resistance to *C*. *albicans* infection can be achieved by Th1/Th17 directed cell-mediated immunity. Th17 differentiation protects against disseminated candidiasis [9].

CD47 is a unique Ig superfamily member that is expressed on many cell types [10,11]. CD47 is a transmembrane receptor that mediates self-recognition, preventing clearance by phagocytic cells, which recognize CD47 via the counter-receptor SIRPα [12]. Several viruses encode cell surface proteins similar to CD47 that enable infected cells to evade host immunity by engaging SIRPα [13]. Similarly, elevated CD47 expression by some cancers protects them against natural killer (NK) cells, macrophage clearance, and antibody-dependent tumor cell killing [14–16].

A second function of CD47 is to support cell migration via its interactions with a subset of integrins and with SIRPγ [11]. CD47 expression on leukocytes and endothelium is thereby critical for immune cells to localize to a site of infection [11,17] and for T cell activation [18]. Blocking surface CD47 using monoclonal antibodies significantly reduced polymorphonuclear (PMN) leucocyte transmigration through collagen coated filters [19]. *cd47* ^-/-^ mice are more susceptible to gram negative bacterial infections due to delayed neutrophil recruitment and a weakened integrin-dependent oxidative burst response [20–22]. Furthermore, *E*. *coli* K1 up-regulated CD47 on dendritic cells in a mouse meningitis model, thereby increasing tolerance to infection [23]. In contrast, *cd47* ^*-/-*^ mice are less susceptible to developing *Staphylococcus aureus*-induced arthritis [24] and to LPS-triggered acute lung injury and *E*. *coli* pneumonia. PMN recruitment in *cd47* ^-/-^ mice was not impaired in zymosan-induced peritonitis [25], suggesting that recruitment in response to fungal pathogen-associated molecular patterns (PAMPs) does not depend on CD47.

The third important function of CD47 in immunity is to serve as a receptor for the matricellular protein thrombospondin-1 (TSP1). TSP1 binding to CD47 inhibits T cell activation [26–28] and nitric oxide signaling in T cells [29] and increases T cell differentiation into Treg cells [30]. TSP1 signaling via CD47 also inhibits dendritic cell activation, survival, and IL-12 production [31–33]. We recently reported that *thbs1*-/- mice are more resistant to local and disseminated candidiasis [34]. Resistance was associated with more rapid clearance of kidney colonization, enhanced macrophage phagocytosis of *C*. *albicans*, and a more balanced inflammatory cytokine response. TSP1 utilizes several signaling receptors on innate and adaptive immune cells [27,35], so the role of CD47 in mediating these effects of TSP1 on *C*. *albicans* pathogenesis is unclear.

Based on its known immune functions, CD47 could play both protective and sensitizing roles in fungal infections. Because antibodies that block CD47 are entering clinical trials for treating cancer patients [36], it is important to know whether this treatment could increase their susceptibility to disseminated candidiasis. We show that, in contrast to its ligand TSP1, CD47 supports protective immunity against disseminated candidiasis in a mouse model by limiting host pro-inflammatory cytokine/chemokine expression and by limiting neutrophil infiltration.

## Materials and Methods

### Ethics statement

Experimental protocols, housing, and care of mice were conducted in an AAALAC approved facility according to animal study protocol LP-022 approved by the National Cancer Institute Animal Care and Use Committee.

### Strains and growth conditions

For challenge of mice, *C*. *albicans* strain SC5314 [37] was grown overnight in 50 mL of Yeast Peptone Dextrose (YPD) medium at 30°C with aeration as previously described [38]. Cells were harvested by centrifugation at 3,000 g for 10 min, washed twice with 50 ml of sterile non-pyrogenic normal saline (Quality Biological Inc., MD), and resuspended in 10 ml of saline before quantification using a Petroff-Hausser counting chamber. The cell suspensions were adjusted to the final concentration for parenteral administration using non-pyrogenic sterile saline.

### Mouse infection with *C*. *albicans*

Inbred 8 to 12 week old (20–25 g) wild type (WT) and *cd47* ^-/-^ mice on a pure C57BL/6J background (The Jackson Laboratory) were bred in a NCI vivarium and used for all animal experiments. *cd47* ^-/-^ mice were periodically back-crossed against WT to minimize phenotypic drift. Mice were randomly allocated to groups of five animals per cage and provided *ad libitum* access to filtered water and standard mouse chow. Each group of mice was inoculated intravenously in the lateral caudal tail vein using a 30 gauge needle with a volume of 0.1 ml of saline containing 5x10^5^ *C*. *albicans* cells [38,39]. Clinical signs of illness in each mouse were evaluated three times daily, and mice displaying arched backed posture, sunken eyes, ruffled hair or dehydration were euthanized immediately by CO_2_ inhalation and processed for complete necropsy and collection of tissues for histopathological examination. To examine virulence in the absence of CD47, we used 8–10 mice per group. As controls we used WT and *cd47* ^-/-^ mice administered with 0.1 ml of non-pyrogenic sterile saline (n = 5 per group).

To longitudinally monitor effects of CD47 on organ burden and host immune responses, mice infected with *C*. *albicans* were euthanized sequentially from 1 to 6 days post-inoculation (PI). A total of 18 mice from each strain were inoculated with SC5314, and 3 control mice received no fungal challenge. Three animals from each group were sacrificed daily from day 1 to day 6 PI for histopathology and cytokine assays. The 3 control animals, i.e., untreated and uninfected, administered with 0.1 ml of non-pyrogenic sterile saline were sacrificed, and the organs and serum were collected. The mean results for these 3 control animals were used as time zero values. Sera were stored at −80°C until analysis.

Flow cytometric analysis was used to quantify the inflammatory response in spleens, kidneys and brains from WT (n = 5) and *cd47* ^*-/-*^ (n = 5) mice at day 1, 2, 3, 4 and 7 PI with *C*. *albicans* as compared with uninfected control mice. The same experiment was reproduced twice. We analyzed leukocytes infiltration in these tissues at same PI intervals using at least 4 mice per group.

### Necropsy and histopathology

Immediately after euthanasia, macroscopic changes were recorded, and the brain, heart, lungs, liver, spleen, and right kidney were immersed in buffered 10% formalin, processed for paraffin embedding, sectioned at 5 μm, and stained with H&E. Grocott’s modification of Gomori’s methenamine silver (GMS) stain was used for detection of fungi *in situ* [40]. Inflammatory reactions were scored between ++++ for severe inflammation with PMN infiltration and + for localized inflammatory foci.

Histopathology images from sections of formalin-fixed and paraffin-embedded tissues stained with Gomori's methenamine silver or H&E were obtained using a light microscope (Olympus BX51) fitted with a digital camera (Nikon DXM1200F) and ScanScope XT digital scanner (Aperio). Images were processed with Adobe Photoshop and Aperio ImageScope v11.1.2.760 (Aperio).

### Organ burden quantification

Three mice from each group were euthanized at day 1, 4 and 7 PI to determine the fungal burden in their kidneys. After sterile isolation, kidneys were weighed and homogenized in 1.0 ml of nonpyrogenic sterile saline. 10 fold serial dilutions of 10^−2^, 10^−4^ and 10^−6^ in 0.1 ml of the homogenates were spread on triplicate on plates containing Nickerson’s medium, also known as BiGGY agar, a selective and differential medium for *C*. *albicans* [41]. After 48 h of incubation at 30°C, colony number, morphology, and color were recorded, and numbers of CFU per kidney were estimated. *C*. *albicans* appears as brown to black colonies with no pigment diffusion and no sheen [41].

### Determination of serum cytokines and chemokines

Serum was collected from sacrificed mice at various time points following infection with *C*. *albicans* in WT and *cd47* ^-/-^ mice. A Luminex bead array Milliplex MAP Kit (catalog no MPXMCYTO-70K, Millipore, Billerica, MA) was used to quantify cytokines including IL-4, IL-6, IL-10 IL-17, and TNF-α, according to the manufacturer's specifications.

### Mononuclear cell isolation

Single cell suspensions from brain and kidney were prepared after intracardiac perfusion of anaesthetized mice with 20 ml of normal saline to remove the circulating blood lymphocytes. Brain tissues were incubated with 1 ml of collagenase D (1 mg/ml; Roche) at 37°C for 30 min followed by mechanical disruption of the organs through a 100 μm filter. The homogenates were resuspended in 4 ml of 90% Percoll (GE Healthcare) in HBSS, and a gradient was prepared by overlaying 3 ml of 60% Percoll, 4 ml of 40% Percoll and 3 ml of HBSS respectively. The gradients were then centrifuged at 1,700 rpm for 18 min at 4°C, after which the band corresponding to mononuclear cells was isolated, and single cell suspensions were washed with HBSS and then resuspended in RPMI 1640.

Kidney tissues were mechanically disrupted with a 100 μm filter, after which the homogenates were resuspended in 8 ml of 40% Percoll, and gradients were prepared by overlaying the cell suspension on top of 70% Percoll (3 ml) in 15 ml tubes [42]. The gradients were centrifuged at 2,000 rpm for 30 min at 4°C, after which the band of mononuclear cells present at the 70%-40% interface was isolated and subsequently washed to make single cell suspensions. In all cases, the absolute number of mononuclear cells from each organ was determined prior to flow cytometry analysis. Splenocytes were prepared by mechanical disruption of spleens on 100-μm filters, after which the cells were treated with red blood cell lysis buffer (0.14 M NH_4_Cl and 0.017 M Tris-HCl, pH 7.2), and washed twice before staining. In all cases, the absolute number of mononuclear cells from each organ was determined prior to flow cytometry analysis.

### Flow cytometry

Mononuclear cells isolated from different organs were blocked with 3.3 μg/ml of anti-mouse CD16/CD32 (Fc block; BD Biosciences) in PBS containing 1% FBS for 20 min prior to antibody staining. The following antibodies were used to characterize leukocyte populations: CD45.2-FITC (BD Bioscience Clone 104), CD8-Pacific Blue (Invitrogen, Clone MH10), CD4 Qdot 605 (Invitrogen, Clone RM4-5), CD11b-PE-Cy7 (eBioscience, Clone M1/70), Gr1-APC (BD Bioscience, Clone RB6-8C5), CD11c-APC-Cy7 (Biolegend, Clone N418), Thy1.2 Alexa Fluor-700 (Biolegend, Clone 30/H12), NK1.1 PerCP Cy5.5 (BD Bioscience, Clone PK136), CD4-PeCy7 (Biolegend, GK1.5), and CD45.2-APCy7 (Biolegend, Clone 104). The primary antibody staining was performed for 30 min on ice in 100 μl of FACS buffer (PBS containing 1% FBS), the cells were analyzed using flow cytometry (Digital LSR II; BD), and the data were analyzed using FlowJo software (Tree Star, Inc).

For intracellular flow cytometry (IC flow) experiments, splenocytes were harvested from cohorts of WT and *cd47* ^-/-^control and infected mice at 7 days PI. Single cell suspensions of splenocytes were stimulated with PMA/ionomycin (Sigma) for 2 h at 37°C followed by 2 h incubation in the presence of Golgi stop and Golgi plug (BD bioscience). Intracellular staining was performed to measure cytokine profiles of T cells from non-infected and infected mice. Briefly, splenocytes were treated with fixation buffer (eBioscience) for 30 min and then washed twice with permeabilization buffer. Cells were then stained with CD4-PE-cy5 (RM4-5), IL4-FITC(11B11), IL17-APC (eBio17B7), IFNγ-PE (XMG1.2), and Foxp3-PB (MF-14), incubated at 4°C for 30 min, washed, resuspended in PBS+0.1% BSA+0.01% azide and analyzed using LSR II and FACS Diva software.

### Collagen deposition analysis

To estimate levels of fibrosis, unstained 3 μm-thick kidney sections obtained from paraffin blocks were deparaffinized, rehydrated, and placed in 0.2% phosphomolybdic acid for 2 min to reduce nonspecific staining. Sections were stained with 0.1% Sirius red (Polysciences Inc., Washington, DC) in saturated aqueous picric acid for 1 h and dipped in 0.01% HCl for 30 s for differentiation. The slides were immediately dehydrated and mounted with cover slips. Collagen fraction was determined by measuring the area of stained tissue with Quantitative Morphometric analysis. Three representative micrographs at 200x magnification of each mouse Sirius red stained kidney sections were analyzed within a given field, expressing that area as a proportion of the total area under observation using Adobe Photoshop. Each time point represents the average of nine micrographs of *Candida* colonized kidneys. A threshold grey level for stained collagen was calculated as a percentage to total area [43].

### ELISA for anti-mannan IgM

Wells of 96-well microplates were coated with 50 μl of anti-mouse IgM in DPBS (for standard curve) or mannan (Sigma) at 25 μg/ml in 100 mM sodium carbonate, pH 9.2, and incubated overnight at 4°C. Plates were washed three times with 200 μl of DPBS/0.05% Tween-20 and blocked with 1% BSA/DPBS at room temperature for 30 min. After washing, plates were incubated overnight at 4°C with 100 μl of sera diluted 1/100 in 1% BSA/DPBS. After washing, the plates were incubated with 100 μl/well of 2 μg/ml biotinylated anti-mouse IgM (R6-60.2) at RT for 1 h.

The plates were washed six times and then incubated with 100 μl of 1/1000 dilution of avidin-HRP in 1% BSA/DPBS at RT for 30 min. After washing six times, 50 μl/well of OPD (o-phenylenediamine, Sigma) in 0.05 M phosphate-citrate buffer/0.03% sodium perborate pH 5 were added. After 7–10 min incubation, color development was stopped by addition of 100 μl per well of 3 M sulfuric acid, and the optical density was measured at 490 nm.

### BMDM culture and phagocytosis and viability assays

To make primary bone marrow-derived macrophages (BMDMs), age- and gender-matched mice were sedated using isoflurane and euthanized with cervical dislocation. All hind leg bones were removed, cleaned of all tissue and sterilized with 70% ethanol. Femurs and tibias were flushed with RPMI II supplemented with L-Gln. Red blood cells were lysed with ACK Lysis Buffer. Cells were plated and differentiated into macrophages using 30% L929 conditioned medium in RPMI supplemented with L-Gln, Pen-Strep, and 10% FBS as described previously [44]. L929 cells were a kind gift from Dr. Alan Sher, NIH. BMDMs were lifted using EDTA, counted and plated.

The phagocytosis assay was adapted from a published method [45]. Briefly, differentiated BMDMs from wild type and *cd47* ^*-/-*^ mice were incubated in OptiMEM for 2.5 h and lifted with EDTA. Macrophages were added to polypropylene tubes (USA Scientific) containing 0, 0.5, 1, or 5 multiplicity of infection (MOI) of either wild type or GFP^+^ *C*. *albicans* strain SC5314 (provided by Dr. Judith Berman) [46] in OptiMEM, and incubated with shaking for 15 min or 30 min as indicated. Unbound yeast were washed from the BMDM using ice cold FACS Buffer (PBS containing 3% fetal calf serum), and the remaining cells and yeast were stained with anti-*Candida* primary antibody (AbCam, ab53891) and secondary goat anti-Rabbit APC (Invitrogen). Fluorescence was detected using a BD LSR II flow cytometer and analyzed with FlowJo (Tree Star Inc). Cell viability was assessed by lactate dehydrogenase (LDH) release following manufacturer’s instructions (Promega).

For the i*n vitro* killing assay hind leg bones from WT (N = 4) and *cd47* ^*-/-*^ *(N = 4)* mice were removed and flushed with cold HBSS+0.5% BSA+2 mM EDTA. Red blood cells were lysed with ACK lysis buffer. Neutrophils or macrophages were isolated by negative selection using neutrophil and macrophage isolation kits (Miltenyi Biotec) following the manufacturer’s instructions. In short, cells were incubated with depleting biotin-antibodies, followed by magnetic anti-biotin beads and magnetic separation. Macrophages were grown for 3 days in DMEM/F12 supplemented with L-Gln, Pen-Strep, 10% FBS, and 10 nM mouse recombinant M-CSF (eBioscience). Before inoculation, neutrophils or macrophages were washed 3x in serum-free RPMI and plated in a 96 well plate. Neutrophils or macrophages were inoculated with 1x10^5^ *C*. *albicans* in serum-free RPMI in duplicate. After 4 hours, a serial dilution of the cells was plated on YPD plates for 30 h at 30°C in duplicate. The colonies formed were counted and compared to the non-inoculated control.

### Inflammatory gene expression

Inflammatory gene expression analysis was done in WT and *cd47* ^-/-^ mice at day 3 PI. Specific mRNA levels in total kidney and spleen RNA were analyzed by NanoString methodology as previously reported [47,48] and conducted at the DNA sequencing core facility of NIH. Briefly, 100 ng of total RNA per kidney were hybridized to the target specific mouse inflammatory gene CodeSet at 65°C. The CodeSet contained probes against a panel of 179 genes encoding proteins involved in mouse inflammation and six internal reference genes and were used to analyze local inflammatory response in kidneys. The hybridized reactions were loaded onto the NanoString Prep station, which removes excess reporter, binds the reporter to the cartridge surface, and stretches the probes for scanning. Subsequently, the cartridges were loaded onto the NanoString Digital Analyzer and scanned. This method provides a quantitative analysis of gene expression [49–51]. Complete primary data is available in S1 Data. qRT-PCR was used to validate selected NanoString data, using at least three mice per replicate. Primers used for qRT-PCR are listed (Table 1).

**Table 1:** Primers used for real-time PCR analysis of mRNA expression.

	Antisense (5'-> 3')	Sense (5'-> 3')
Nlrp3	AGCCTACAGTTGGGTGAAATG	CCTACCAGGAAATCTCGAAGAC
ASC	TCACAGAAGTGGACGGAGTG	TCATCTTGTCTTGGCTGGTG
Naip5	GTCAATGTGTGTCCCGACTG	TGGGGATGTCTTTCCTTCAC
NLRC4	ACGCAGGCAAAACACTCATA	TCGTTTCTCAAGCCAATTCC
NOD2	AACATCAGGCAGAATCCCTCT	ACCAACCATCACGACTCCTC
AIM2	AACCCAAGCAAAACAAAGTG	GCTACAAGGTCCAGATTTCAAC
Caspase-1	ATGCCGTGGAGAGAAACAAG	GGTGTTGAAGAGCAGAAAGCA
NLRP1	GAGACCACAAACCAAGACAAGA	ACAGAGACCCCACCCAACT
IL-1b	TGGGCTGGACTGTTTCTAATG	TTTCTTGTGACCCTGAGCG

The Excel-based method described by the manufacturer or the delta-delta Ct method was used to perform normalization compared to six internal controls and basic statistical analysis of the data. The normalized results are expressed as the relative mRNA level, and values for infected WT and *cd47* ^-/-^ kidneys were averaged and shown as mean ± s.d. Statistical significance was calculated using Student's *t* test with significance as p<0.05. Using Genego software in MetaCore [48], up- and down-regulated genes clusters were analyzed for significant pathways.

### Statistics

The probability of survival as a function of time was determined by the Kaplan-Meier method, and significance was determined by the log-rank (Mantel-Cox) test and Jehan-Breslow-Wilcoxon test using GraphPad Prism software. Serum cytokine expression patterns and flow cytometry data among all treatment groups at various time points were analyzed by two-way ANOVA with post Bonferroni comparison test. Three to 4 randomly selected mice from each group were euthanized at each time point for longitudinal comparisons. Data were analyzed for significant differences by comparing means of each triplicate reading at various time points assuming that the cytokine expression levels within each group of mice are normally distributed [52]. Collagen fraction was analyzed using Student’s *t* test. Intracellular flow cytometry analysis was done by using unpaired Student *t* tests.

## Results

### CD47 limits lethality in murine candidiasis

**Fig 1:**
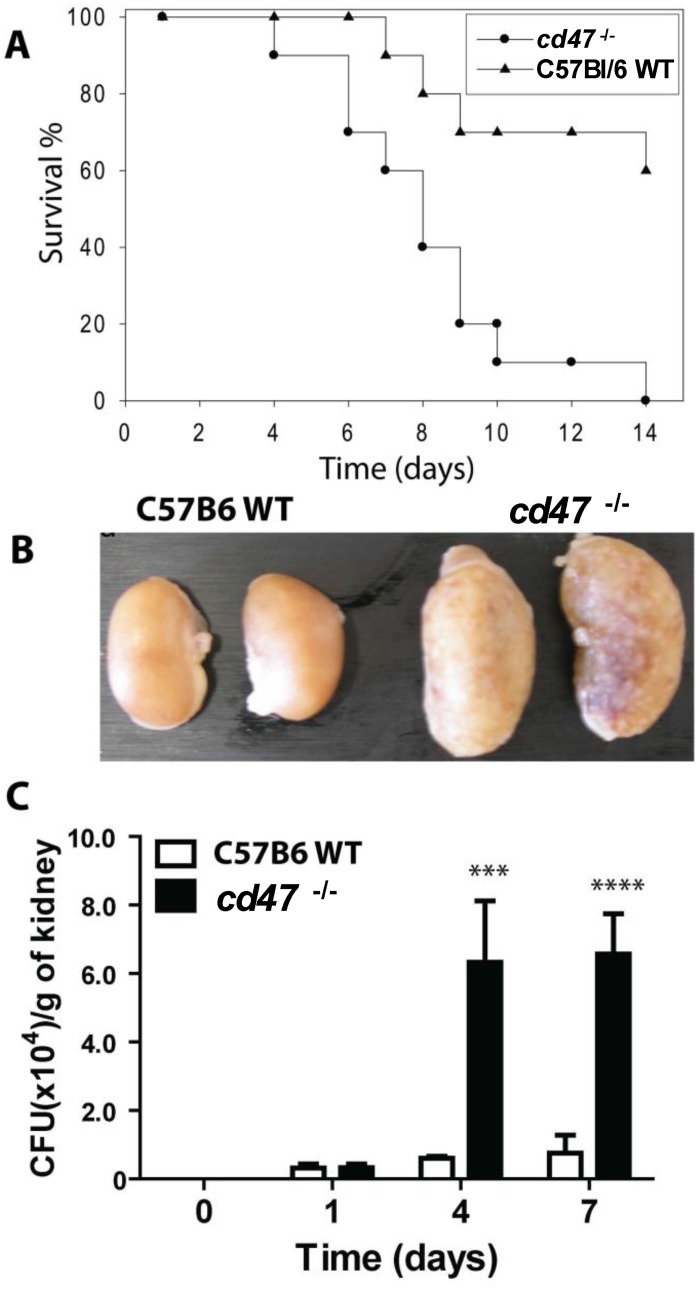
*cd47*-null mice are more susceptible to disseminated *Candida* infection. **(A)**. Each group consisted of fifteen 8–12 week old female mice. Mice were administered 5x10^5^ cells through the lateral tail vein. Results were analyzed using log-rank (Mantel-Cox) test and Jehan-Breslow-Wilcoxon test. (B). Kidneys of mice infected with *C*. *albicans*. WT mice did not show gross lesions, whereas CD47 null mice showed enlarged mottled kidneys with superficial micro-abscesses. (C). Kidney fungal burdens of mice infected with *C*. *albicans* SC5314 strain. Solid bars represent mean CFU of infected left kidney homogenates of *cd47* ^-/-^ mice determined by CFU on BiGGY agar from six representative serial dilutions from each kidney, representing five mice per time point infected. Open bars represent mean ± SE, n = 3, for WT mice infected with *C*. *albicans* and were analyzed using two-way ANOVA with post Bonferroni comparison test.

An initial experiment using 8 mice per group showed that WT mice infected with 5x10^5^ *C*. *albicans* had significantly higher survival compared with infected *cd47* ^-/-^ mice (p <0.002, hazard ratio estimate of 9.0 with 95% confidence interval 2.2–36.6). These results were reproduced in a second experiment with 15 mice per group (hazard ratio estimate of 7.1 with 95% confidence interval 2.1–23.4, P <0.001, Fig 1A). *cd47* ^-/-^ mice inoculated with *C*. *albicans* died as early as 4 day post-infection (PI) and suffered 100% mortality by 14 days PI. Infected WT mice did not die until 7 day PI, and 60% survived at the end of the experiment on day 14 PI. Control mice administered with intravenous saline alone had no mortality (data not shown).

### CD47 alters pathogenesis of candidiasis in kidney and brain

Kidney is the primary initial colonization site in disseminated candidiasis. We observed striking difference in gross pathology in the kidneys of infected mice. *cd47* ^-/-^ mice had enlarged kidneys compared with the WT during the entire course of infection except day 1 PI. Fig 1B shows a representative picture at day 4 PI, where infected *cd47* ^-/-^ kidneys were twice as large as infected WT, suggesting more edema and inflammation. In a longitudinal study, CFU in the kidney was comparable in WT and *cd47* ^*-/-*^ mice at day 1 PI but significantly higher in *cd47* ^*-/-*^ mice at days 4 and 7 PI (p <0.001 and 0.0001 respectively, Fig 1C).

**Fig 2:**
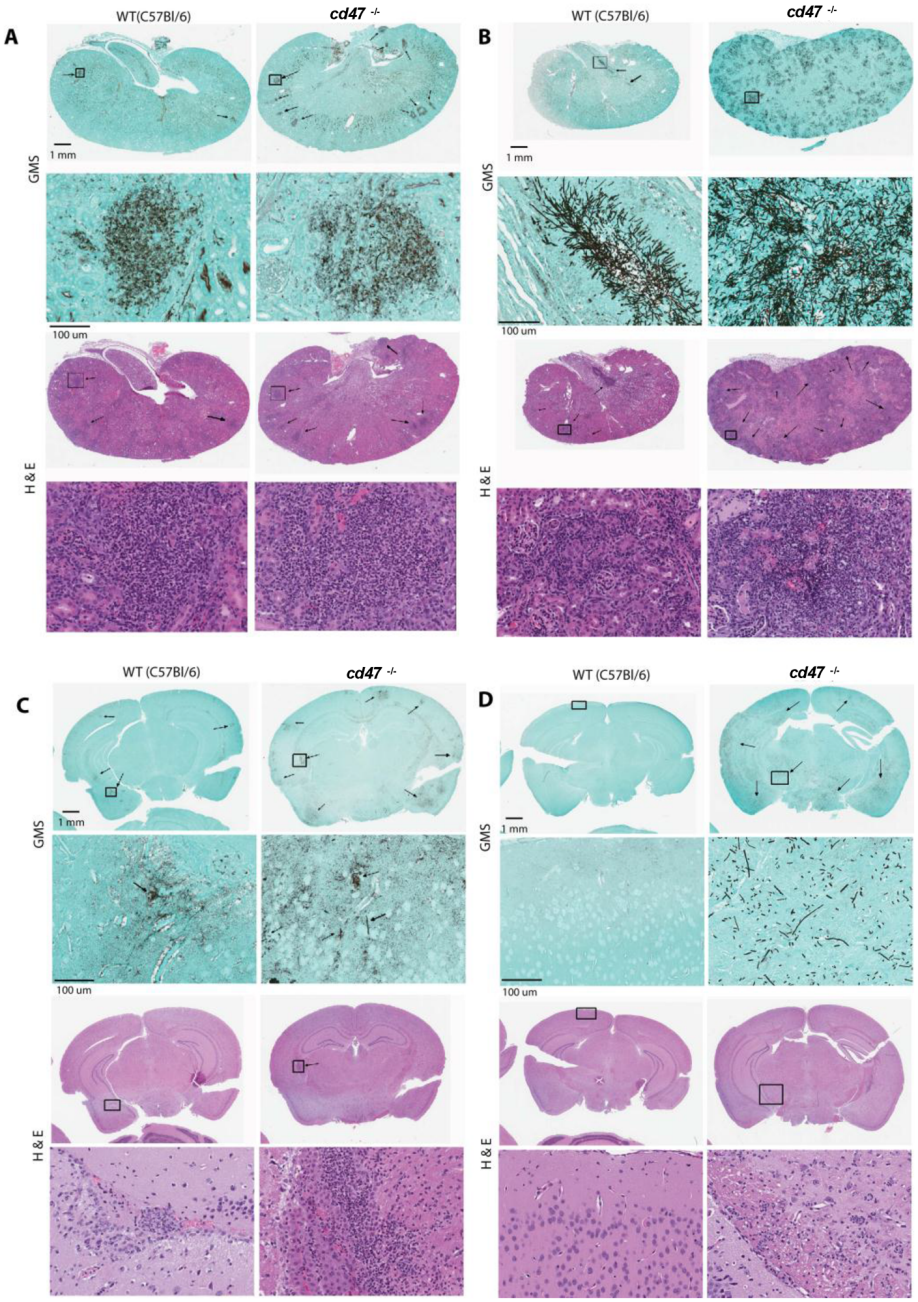
Kidneys and brains of *cd47* ^-/-^ mice infected with *C*. *albicans* show increased fungal colonization and inflammatory responses. (A & C) Representative GMS-stained sections of mouse kidney and brain tissue to detect *C*. *albicans* colonization (black) and H&E stained sections to show inflammatory reactions in *cd47* ^-/-^ kidneys compared with the WT at day 2 PI. (B & D) Comparative histopathology of mice with disseminated candidiasis at 4 days PI. Kidney and brain tissues were stained using GMS to detect *C*. *albicans* colonization (black) and H&E to assess inflammatory reactions. *cd47* ^-/-^ kidneys showed higher fungal colonization and more inflammation. *cd47* ^-/-^ brains showed higher fungal colonization at both time points.

Inflammatory response and fungal colonization was further assessed using H&E and fungal-specific GMS stained sections (Fig 2). Histopathological examinations did not show differential colonization by *C*, *albicans* or immune responses until 4 days PI. At 4 days PI *cd47* ^*-/-*^ kidneys exhibited an increased inflammatory response. Representative kidney sections show similar extent and distribution patterns of *C*. *albicans* colonization and inflammatory response at 2 days PI (Fig 2A). Consistent with the CFU data in Fig 1, C. *albicans* colonization became greater at 4 days PI in the *cd47* ^-/-^ mice compared to wild type (Fig 2B). *cd47* ^-/-^ mice had more progressive colonization in kidneys compared with the WT mice. *C*. *albicans* broadly colonized the kidneys of *cd47* ^-/-^ mice, whereas WT mice had focal colonization (Fig 2A and 2B GMS sections). Inflammatory changes assessed on H&E sections followed a similar pattern (Fig 2A and 2B H&E sections). The kidneys of *cd47* ^-/-^ mice showed more abundant immune cell infiltration and tissue necrosis. At later times, we consistently observed increased inflammation in the kidneys of *cd47* ^-/-^ mice (data not shown).

The pattern of colonization in the brain mirrored that observed in kidneys, with *C*. *albicans* disseminating more broadly in brains of *cd47* ^-/-^ mice (Fig 2C & 2D). However, in contrast to kidneys, we did not notice major inflammatory changes in the brains of *cd47* ^-/-^ mice (Fig 2C and 2D H&E sections). Interestingly, WT brains showed very little colonization of *C*. *albicans* associated with local inflammatory reactions. Overall, *cd47* ^-/-^ mice exhibited more fungal invasion and colonization in both kidney and brain, but inflammatory reactions differed in these organs.

### CD47 alters serum cytokine responses during systemic candidiasis

**Fig 3:**
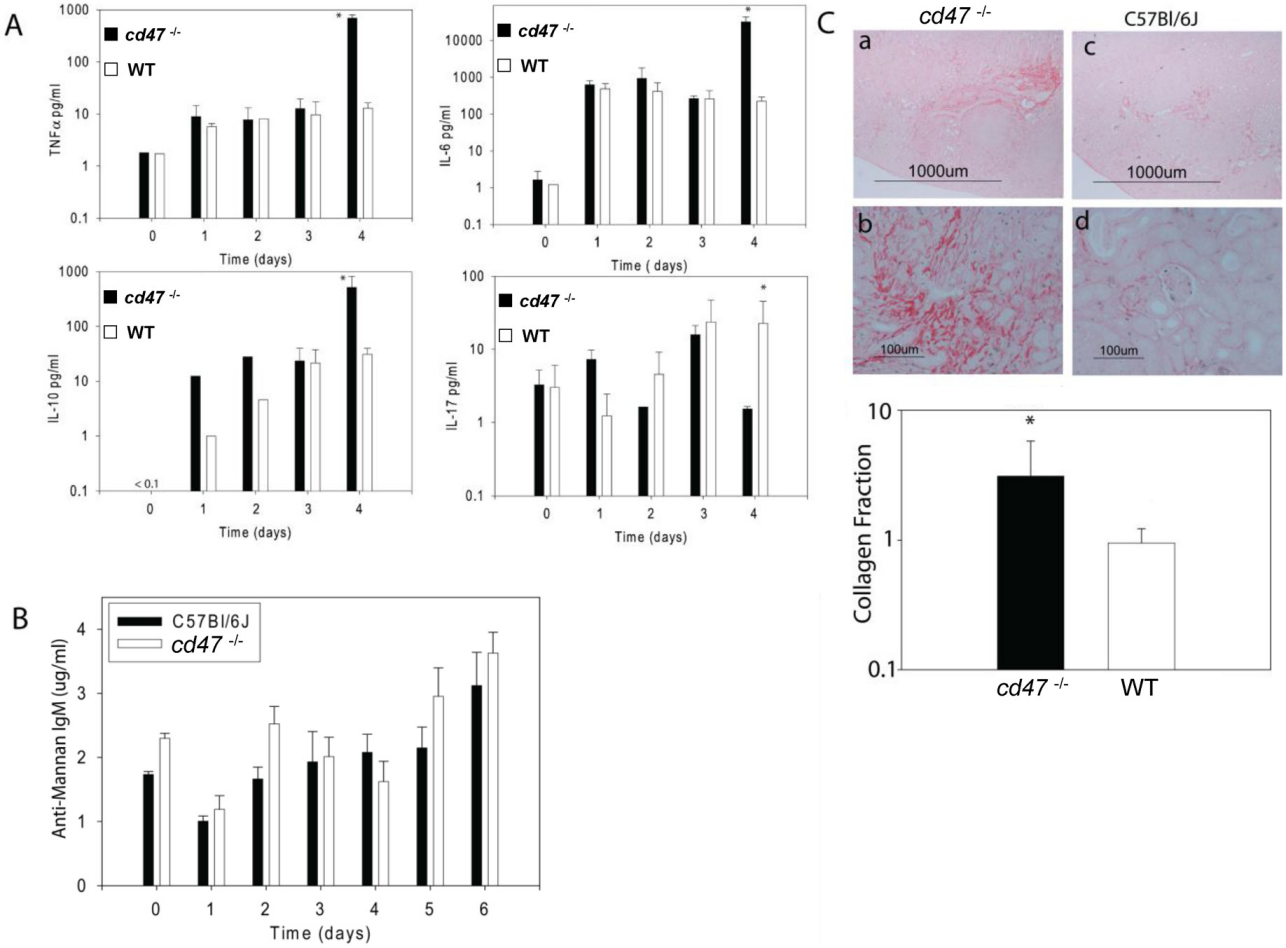
Inflammatory responses in WT and *cd47* ^-/-^ mice associated with disseminated candidiasis. (A) Cytokine levels were compared in mouse sera at the indicated times post inoculation. 4 days PI is the crucial time for *cd47* ^-/-^ mouse mortality at this inoculation dose. This point is associated with higher TNFα, IL-6 and IL-10 and lower IL-17. Protection against candidiasis in WT mice is associated with higher IL-17 and lower TNFα, IL-6 and IL-10. (B). Mannan-specific IgM levels in sera from infected WT and *cd47* ^-/-^ mice. (C).Collagen staining with Sirius red to assess the level of fibrosis in *Candida* infected kidneys. *cd47* ^-/-^ mice show more fibrosis, consistent with increased kidney failure. Bars represent mean ± SE morphometric quantification for nine representative sections from three mice of each group and analyzed using two-way ANOVA with post Bonferroni comparison test for A and B and Students t test for C.

To examine systemic responses underlying the increased inflammatory responses in *cd47* ^-/-^ mice, we quantified several serum cytokines associated with innate immune responses, up to 4 days PI, when *cd47* ^-/-^ mice started to die (Fig 3A). Consistent with the inflammatory responses and colonization, through 3 day PI we found no significant changes in serum cytokines between the two groups. At day 4 PI, TNFα was significantly increased in *cd47* ^-/-^ mice (p <0.001). Increases in serum IL-6 (p < 0.001) and IL-10 (p < 0.001) levels were also observed in *cd47* ^-/-^ mice. However, IL-17 levels were significantly reduced in *cd47* ^-/-^ mice compared with WT at day 4 PI (p < 0.001). These changes at day 4 PI could be consequences of the divergence in colonization at this time point.

### CD47 does not affect early humoral immunity to *C*. *albicans*

To determine the impact of CD47 on early humoral immunity, we measured mannan-specific IgM levels in serum at different time points PI (Fig 3B). No significant differences in anti-mannan levels were observed between WT and *cd47* ^-/-^ mice at any time point up to 6 days PI. These data suggest that CD47 deficiency does not influence early humoral immunity to *C*. *albicans*.

### CD47 controls kidney fibrosis in disseminated candidiasis

We examined renal interstitial collagen deposition to measure the level of fibrosis in infected kidneys. Infected *cd47* ^-/-^ mice (Fig 3C) showed more Sirius red staining compared with the WT kidneys. Quantitative analyses of representative micrographs showed a 3.0-fold increase in the interstitial collagen volume fraction in *C*. *albicans* infected *cd47* ^-/-^ versus WT kidneys (Fig 3C, P < 0.01).

### Elevated leucocytes infiltration in brain and kidney of infected *cd47* ^-/-^ mice

**Fig 4:**
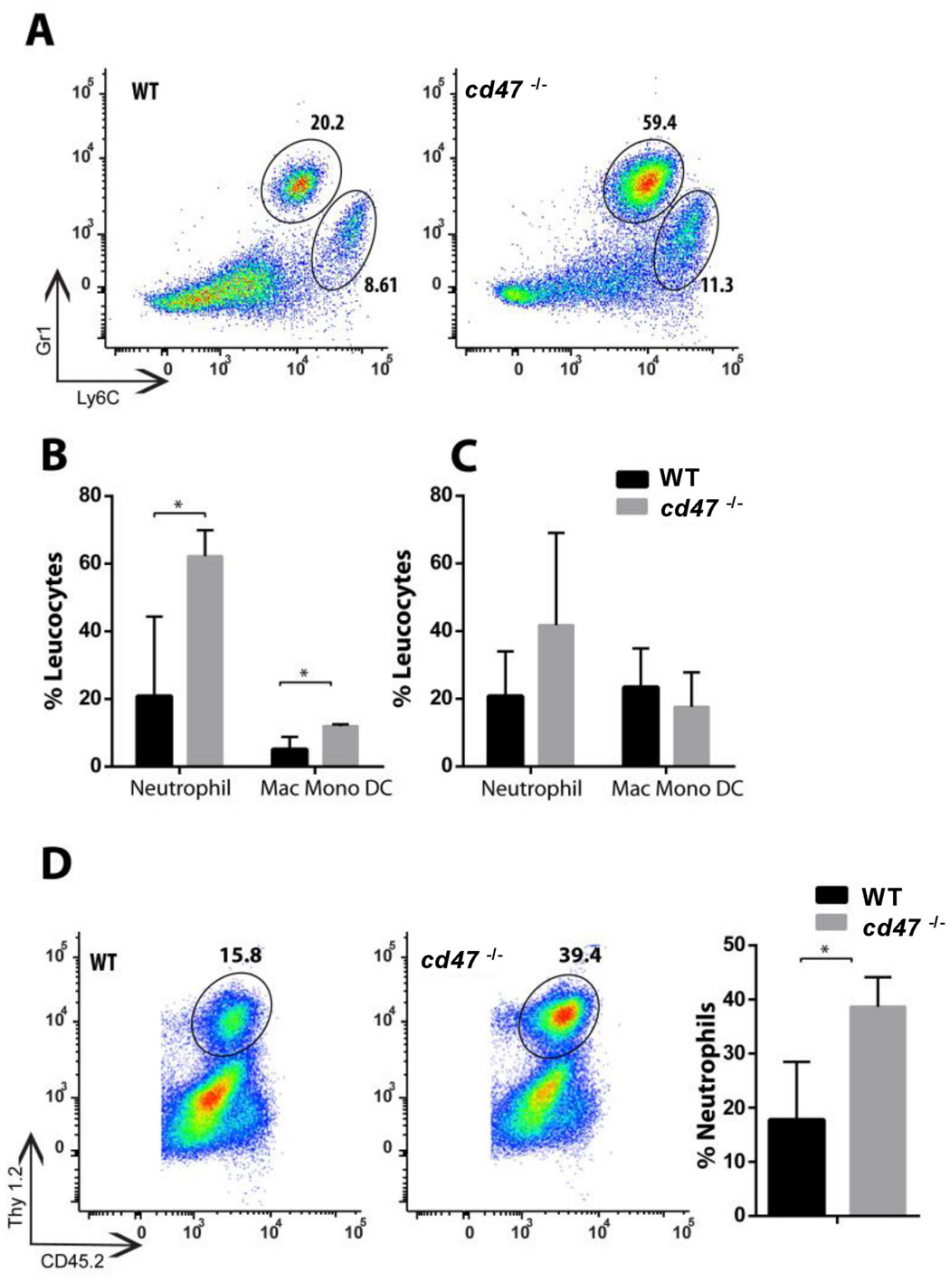
Elevated infiltration of innate immune cells into kidney and brain of infected *cd47* ^*-/-*^ mice. (A) Representative FACS plots showing mononuclear cells infiltration into infected kidney at day 4 PI. Neutrophils are shown on the top circular compartment gated on CD45.2^+^, Thy1.2-ve, CD11b^+^, Gr1^+^ and Ly6c^+^ markers and macrophage, monocytes and dendritic cells in the lower compartment gated on CD45.2^+^, Thy1.2-ve, CD11b^+^, Gr1-ve and Ly6c^+^ markers. (B) Bar graph shows the percentage of infiltrated immune cells at day 4 in candida infected kidney. (C) Bar graph shows infiltrated immune cells in kidney at day 7 PI (n = 5). (D) Representative FACS plot showing T cells (CD45^+^ and Thy 1.2^+^) infiltration in the brain of candida infected animals measured at day 7 PI. The right bar graph shows the percentage of neutrophils (n = 5). Results from 5 mice per group are presented with SE and analyzed using two-way ANOVA with post Bonferroni comparison test.

In order to confirm our histopathology data, we conducted quantitative analysis of mononuclear cell infiltration into brains and kidneys by flow cytometry. At day 1, 2, 3, 4 and 7 PI, we quantified neutrophils, macrophages/monocytes, and T cells in the brains and kidneys of infected WT vs *cd47* ^-/-^ mice. No significant differences were found at day 1–3 PI (data not shown). As expected, at day 4 PI, a statistically significant increase (p<0.05) in neutrophils and macrophage/monocyte/DC populations were observed in the kidneys of *cd47* ^-/-^ mice when compared to WT controls (Fig 4A and 4B). At day 7 PI neutrophil infiltration trended higher in infected *cd47* ^-/-^ mice (Fig 4C). A similar analysis of brain cells showed no differences at days 1 to 4 PI. However at day 7 PI, infected *cd47* ^-/-^ brains showed significantly higher neutrophil infiltration (p <0.05, Fig 4D). No differences in the numbers of CD8^+^ and CD4^+^ T cells were observed between the groups (data not shown). The increased late neutrophil infiltration is consistent with the histopathological findings observed in tissues obtained from infected *cd47* ^-/-^ mice (Fig 2).

### CD47 does not regulate macrophage phagocytic activity or viability

**Fig 5:**
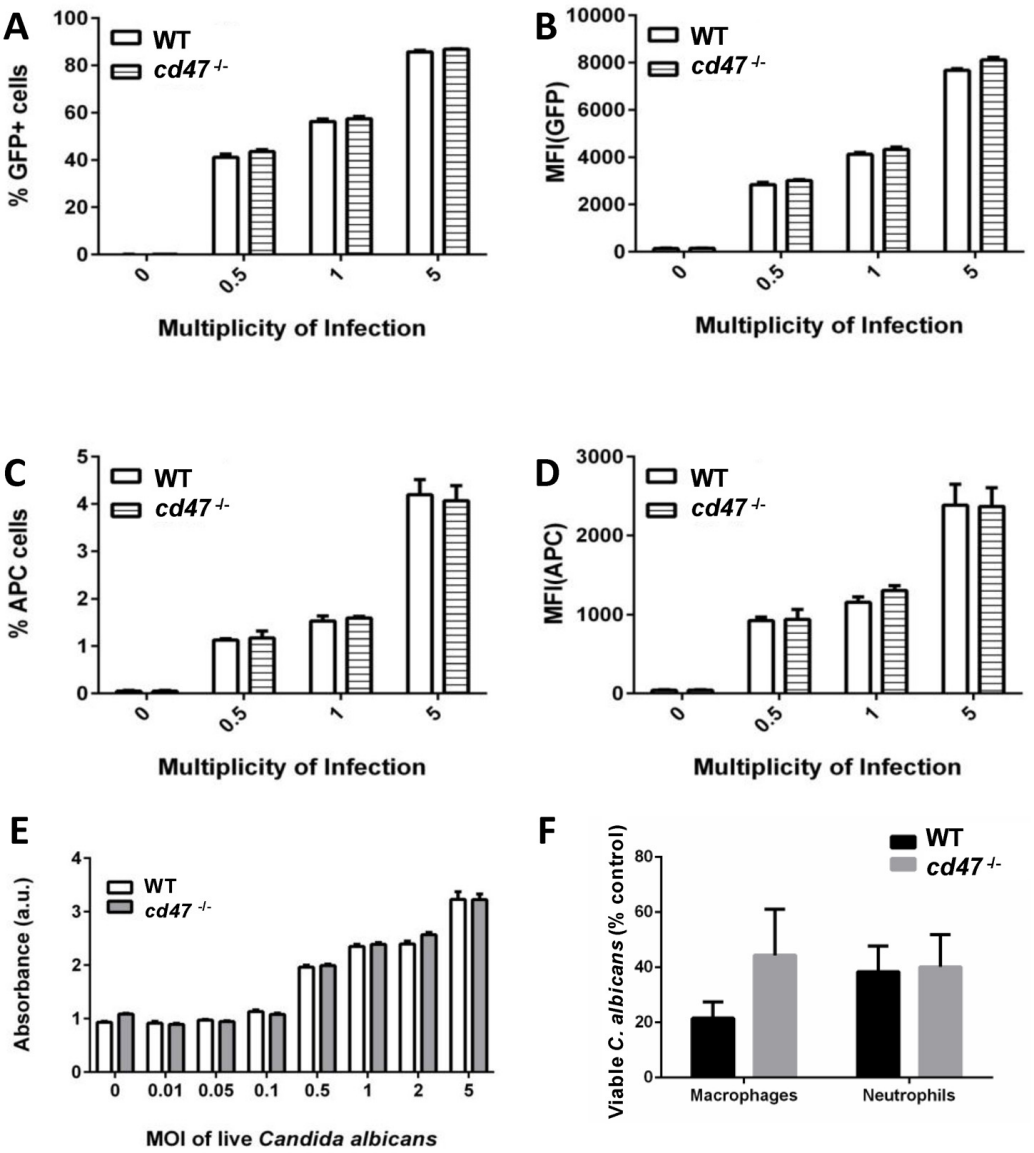
CD47 does not affect macrophage viability, phagocytosis or attachment of *Candida albicans*. Wild type and *cd47* ^*-/-*^ bone marrow was harvested from mice. Bone marrow derived macrophages (BMDMs) were isolated and differentiated. *C*. *albicans* endogenously expressing GFP was incubated with WT and *cd47* ^*-/-*^ BMDMs. Flow cytometry was performed to measure the (A) percent GFP^+^ BMDMs and (B) mean fluorescence intensity (MFI) of GFP. Anti-*Candida* antibody indirectly conjugated to APC was used to stain *C*. *albicans* attached to the outside of BMDMs. (C) Percent of BMDMs expressing APC and (D) MFI of APC was measured. GFP- strain of *Candida albicans* was used to calculate background noise and basal fluorescence. (E) BMDM death caused by incubation with *C*. *albicans* was assessed by release of LDH at the indicated multiplicities of infection (MOI). LDH release from *C*. *albicans* was minimal under these conditions. Experimental data is representative of 3 independent experiments. (F) Killing of *C*. *albicans* by macrophages and neutrophils isolated from WT and *cd47* ^*-/-*^ mice. Results are presented as the percent surviving *C*. *albicans* after 4 h as assessed by colony forming units (mean ± SEM, n = 4). Significance was analyzed by ANOVA.

The above results show increased kidney colonization in *cd47* ^*-/-*^ mice beginning at day 4 PI that occurs despite more accumulation of innate immune cells in this infected organ, which suggests that the absence of CD47 could cause a defect in the phagocytic activity or viability of these cells in the presence of *C*. *albicans*. To address these questions we assessed phagocytic activity of BMDM from WT and *cd47* ^*-/-*^ mice (Fig 5A and 5B). WT and *cd47* ^*-/-*^ BMDM had similar abilities to phagocytose GFP-expressing *C*. *albicans* as assessed by flow cytometry. The smaller number of *C*. *albicans* bound to the surface of the BMDM did not differ between WT and *cd47* ^*-/-*^ cells (Fig 5C and 5D). The ability of *C*. *albicans* to kill macrophages was assessed by LDH release and did not differ between WT and *cd47* ^*-/-*^ BMDM (Fig 5E). Control experiments verified that lysis of *C*. *albicans* by the BMDM did not contribute significantly to the LDH signal. Therefore, the increased kidney colonization in *cd47* ^*-/-*^ kidneys is probably not caused by a defect in the phagocytic activity or viability of *cd47* ^*-/-*^ macrophages in the presence of *C*. *albicans*.

To determine whether neutrophil or macrophage killing of *C*. *albicans* is impaired in the absence of CD47, BMDM and neutrophils purified from bone marrow were assessed for killing of C. albicans in vitro (Fig 5F). No significant differences from killing by the respective WT BMSM or neutrophils were observed.

### Enhanced Th2, Th2 and Th17 responses in infected *cd47*-null mice

**Fig 6:**
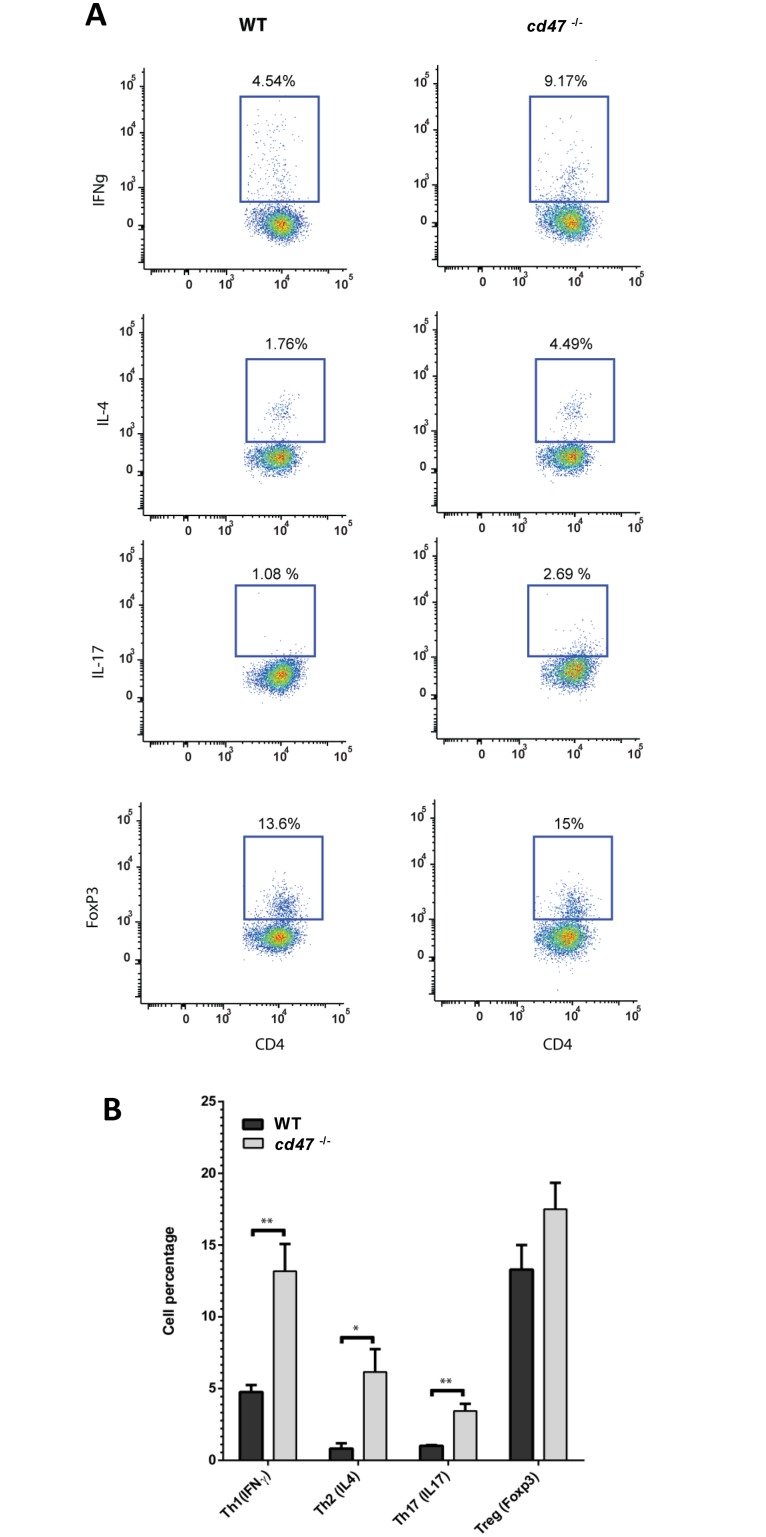
Th subset expansion in spleens of *cd47* ^*-/-*^ and WT mice infected with *C*. *albicans*. Intracellular flow cytometry was performed on splenocytes at day 7 PI. Splenocytes were stimulated with PMA/ionomycin, and cytokine profiles were measured by five color multi-parameter flow cytometry. (A) Representative flow plots of CD4+ T cells from spleens of *C*. *albicans* infected WT vs *cd47* ^*-/-*^ mice analyzed for intracellular IFNγ, IL-4, Il-17 and Foxp3 expression. Uninfected WT and *cd47* ^*-/-*^ mice showed no difference in expression (data not shown). (B) Cumulative bar graph presenting the percentages of Th1 (IFNγ+), Th2 (IL-4+), Th17 (IL-17+), and Treg (Foxp3+) CD4+ T cells in infected WT and *cd47* ^*-/-*^ mice. Results from 5 mice per group were analyzed using two-way ANOVA with post Bonferroni comparison test.

Intracellular flow cytometry was performed to measure Th cell subsets in the effector CD4^+^ T cell population from spleens of infected and uninfected mice. Th1, Th2 Th17 and Treg induction did not differ at days 1 to 4 PI. However, at day 7 PI representative flow plots (Fig 6A), and a quantitative analysis (Fig 6B) revealed significant increases in Th1, Th2, and Th17 subsets in the infected *cd47* ^-/-^ mice. The percentage of Th1 cells producing IFNγ was significantly higher (p<0.01) in infected *cd47* ^-/-^ mice. Similarly, CD4+ cells expressing the Th2-specific marker IL-4 were significantly higher (p<0.05) in infected *cd47* ^-/-^ mice. Infected *cd47* ^-/-^ mice also had significantly more (p<0.01) IL-17^+^ T cells. In contrast, the Foxp3^+^ T cell populations did not differ between infected *cd47* ^-/-^ and WT mice (Fig 6). T helper cell subsets did not differ between uninfected WT and *cd47* ^-/-^ mice (data not shown).

### CD47 alters inflammatory gene expression in kidney and spleen

To gain further insights into the mechanism by which CD47 alters local and systemic immune responses induced by *C*. *albicans* infection, we examined differential gene expression in infected kidneys at 3 days PI to profile a local immune response and in spleens to profile systemic immune response [53]. Of the 193 inflammatory genes tested on the NanoString panel, 18 in spleen and 19 in kidney achieved significance comparing infected *cd47* ^-/-^ versus WT (>1.5-fold change with p <0.05, Table 2, S1 Data).

**Table 2:** NanoString analysis of inflammatory gene expression in spleens and kidneys of *cd47* ^-/-^ and WT mice infected with *C*. *albicans*.

Spleen			Kidney		
Gene	FC	P value	Gene	FC	P value
Jun	1.7	0.042	Ccr2	-7.4	0.039
Cebpb	1.9	0.004	Ccl21b	-4.0	0.055
Il1r1	2.0	0.038	Traf2	-2.9	0.006
C4a	2.1	0.042	Nfe2l2	-2.2	0.007
C1qa	2.2	0.046	Stat1	-2.0	0.032
C1qb	2.2	0.044	Map3k5	-2.0	0.052
Il1a	2.6	0.026	Mapk14	-1.9	0.026
Il9	2.6	0.022	Ppp1r12b	-1.8	0.024
Cfl1	2.6	0.042	Mafg	-1.7	0.040
Ifna1	2.7	0.042	Ly96	-1.6	0.055
Il12a	2.7	0.046	Map3k1	-1.6	0.050
Fos	2.7	0.042	Creb1	-1.5	0.049
Ccl8	3.7	0.031	Tlr5	1.5	0.010
Mef2b	4.1	0.034	Cxcl5	2.8	0.036
C7	4.3	0.012	Cfl1	3.0	0.033
Il10	4.4	0.013	Map3k9	3.3	0.052
Cxcl3	17.2	0.007	Csf2	3.6	0.007
Cxcl2	24.2	0.015	Il23r	5.6	0.031
			Csf3	8.6	0.020

Genes with >1.5-fold difference in mRNA expression between infected *cd47* ^-/-^ and WT tissues and P-values <0.05 are shown. FC: fold change in mRNA expression in infected *cd47* ^-/-^ versus WT.

**Fig 7:**
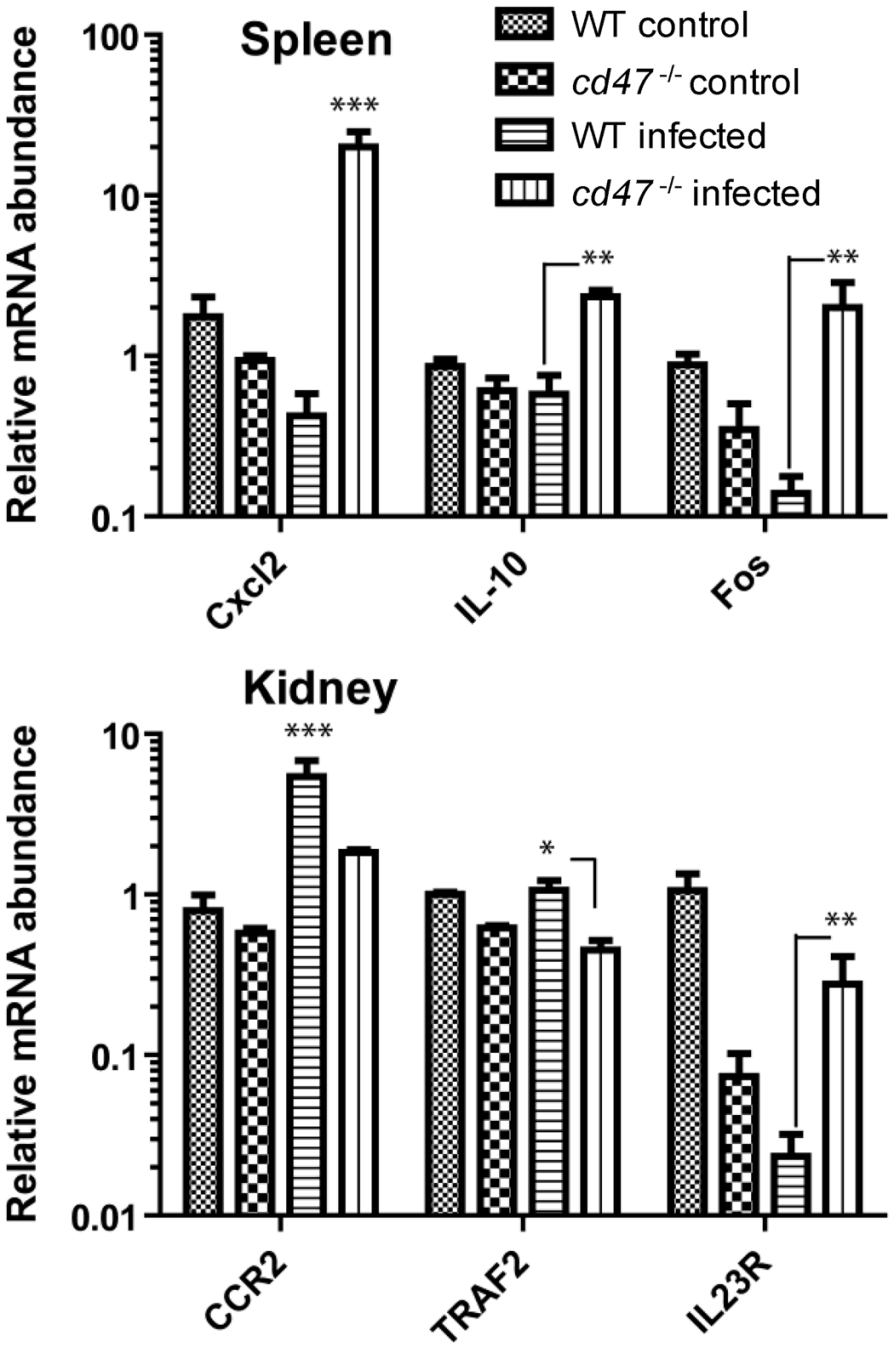
Effect of CD47 on inflammatory gene expression induced by candidemia. mRNA abundance was determined by qPCR using cDNA synthesized from total RNA of spleens and kidneys. Uninfected WT and *cd47* ^-/-^ mice were compared with the infected WT and *cd47* ^-/-^ mice. Each group had at least three mice. Cxcl2, IL-10 and Fos expression in spleens were significantly up-regulated in infected CD47 mice compared with the WT infected mice (p> 0.001, .01 and 0.01 respectively). CCR2 and TRAF 2 of infected *cd47* ^-/-^ kidney tissues were significantly down-regulated compared with the infected WT mice, while IL23R was significantly up regulated in infected *cd47* ^-/-^ mice (p> 0.001, 0.05 and 0.01 respectively). Results from three mice per group were analyzed using two-way ANOVA with post Bonferroni comparison test.

In a separate experiment, we validated our initial gene expression profile by confirming expression patterns of selected genes identified in the NanoString analysis in noninfected WT and *cd47* ^-/-^ vs infected WT and *cd47* ^-/-^ spleens and kidneys (Fig 7). Of the chemokines examined in spleen, Cxcl2 (MIP-2α) and Cxcl3 (MIP-2β) exhibited the highest fold increases in infected *cd47* ^-/-^ vs. WT, while IL-10, C7 (Cxcl10) and Mef2b (myocyte enhancer factor 2B) recorded more than 4-fold increases. Ccl8 (monocyte chemoattractant protein-2) expression was also increased more than 3.5-fold. *cd47* ^-/-^ kidneys also showed over-expression of Csf3 (G-CSF), Csf2 (GM-CSF), Map3k9 (mitogen activated protein kinase kinase kinase 9) and IL-23r, whereas Ccr2 (MIP-1 alpha receptor) and chemokine (C-C motif) ligand 21b (serine) were notably down-regulated in these animals (Table 2 and Fig 7).

**Fig 8:**
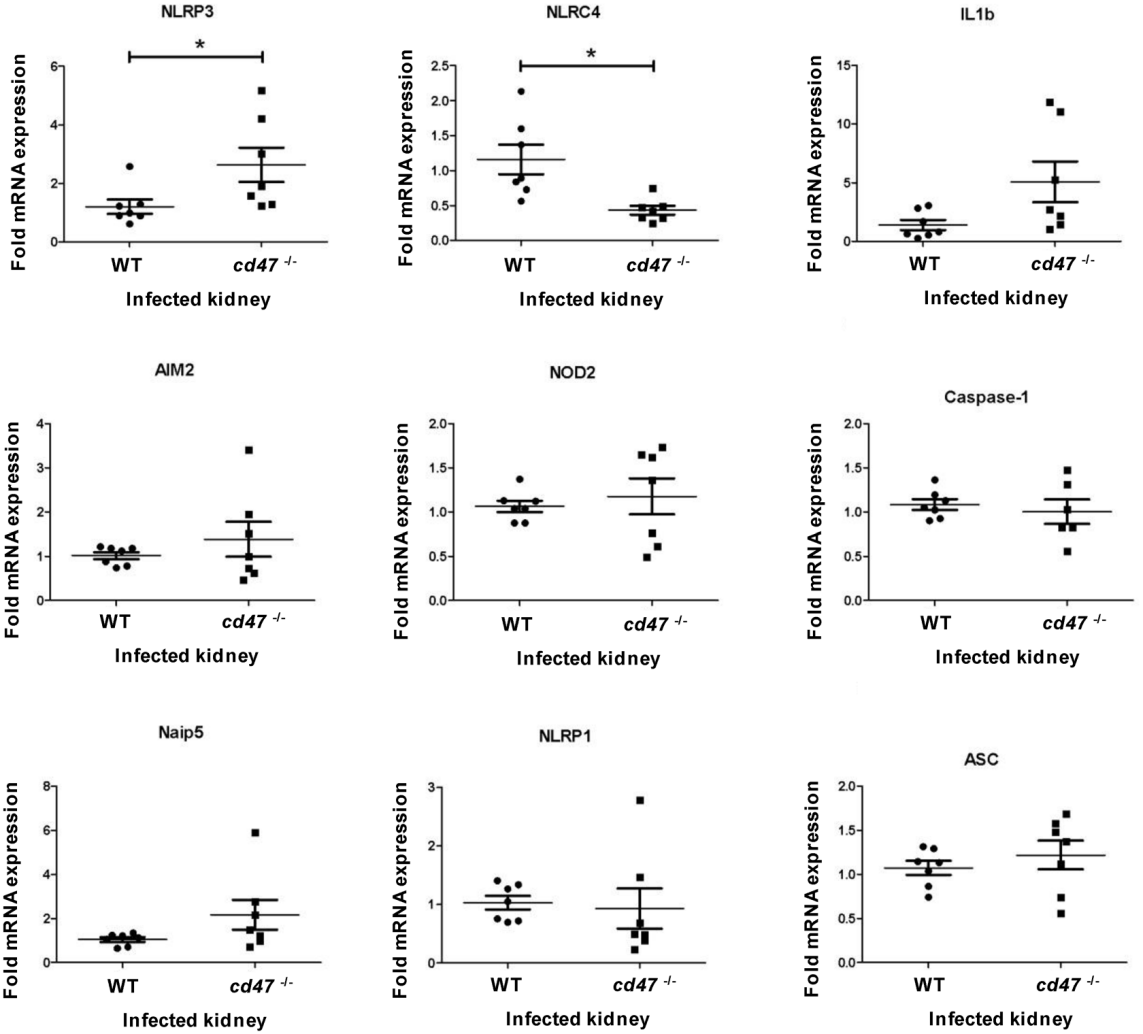
Effect of CD47 on inflammasome pathway gene expression in infected kidney. RNA was purified from kidneys of infected WT and *cd47* ^*-/-*^ mice at 4 days PI. cDNA synthesis was performed, and primers to NLRP3, NCLR4, IL1b, AIM2, NOD2, Caspase-1, Naip5, NLRP1, and ASC were used to quantify mRNA expression levels and normalized to HPRT expression. Each mouse is plotted as individual point, and standard error mean is indicated with a horizontal line. Student’s t test was used to calculate significant (p<0.05) and indicated (*).

To better understand the biological functions affected by these alterations in gene expression, pathway analysis was conducted using MetaCore software. The pathway that achieved the highest significance in spleen was the IL-1 response pathway (Table 3). IL-1α, IL1R1 and Jun (transcription factor AP-1) were up- regulated in the IL-1α pathway. We further examined local regulation of the IL-1 pathway in infected kidneys by assessing mRNA levels for IL-1 and components of the inflammasome pathway involved in its activation (Fig 8). *cd47* ^-/-^ kidneys 4 days PI showed significantly elevated mRNA levels for NLRP3 and decreased levels of NLRC4. IL-1β mRNA was elevated in 3 of the 7 infected kidneys but did not achieve significance for the sample group. Other inflammasome-associated mRNAs including AIM2, NOD2, caspase-1, Naip5, NLRP1, and ASC did not show significant changes in infected kidneys at this time point.

**Table 3:** Analysis of GeneGo pathway maps.

#	*cd47* ^*-/-*^ vs WT spleens	P Value
1	Immune response_IL-1 signaling pathway	7.6E-8
2	Immune response_IL-17 signaling pathways	3.7E-7
3	Immune response_Gastrin in inflammatory response	7.5E-7
4	Mucin expression in CF via IL-6, IL-17 signaling pathways	1.5E-6
5	Immune response_Oncostatin M signaling via MAPK in mouse cells	1.7E-6
6	Immune response_Oncostatin M signaling via MAPK in human cells	2.2E-6
7	Transcription_Role of AP-1 in regulation of cellular metabolism	2.4E-6
8	Immune response_Human NKG2D signaling	2.4E-6
9	Immune response_Murine NKG2D signaling	3.6E-6
10	Development_Ligand-dependent activation of the ESR1/AP-1 pathway	5.5E-6
#	***cd47*** ^***-/-***^ **vs WT kidneys**	**P Value**
1	Immune response_CD137 signaling in immune cell	2.8E-7
2	Cytokine production by Th17 cells in CF	9.4E-7
3	Apoptosis and survival_Lymphotoxin-beta receptor signaling	1.3E-6
4	Cytokine production by Th17 cells in CF (Mouse model)	2.4E-6
5	Immune response_IL-17 signaling pathways	5.4E-6
6	Transcription_Transcription regulation of aminoacid metabolism	1.6E-5
7	G-protein signaling_Ras family GTPases in kinase cascades (scheme)	1.8E-5
8	Apoptosis and survival_Cytoplasmic/mitochondrial transport of proapoptotic proteins Bid, Bmf and Bim	4.1E-5
9	Immune response_Oncostatin M signaling via MAPK in mouse cells	4.4E-5
10	Immune response_Oncostatin M signaling via MAPK in human cells	5.3E-5

Statistics are listed for the top ten statistically significant maps based on differential gene expression in *cd47* ^*-/-*^ versus WT spleens and kidneys generated by MetaCore algorithms.

Pathway analysis of the data in kidney indicated more local up-regulation of the Th17 pathway and down-regulation of apoptosis and survival signaling in immune cells involved in the inflammatory changes in infected *cd47* ^-/-^ mice (Table 3). Signaling between antigen presenting cells and activated CD4^+^ and CD8^+^ through CD137 achieved the highest score. The second most significant pathway that differed in infected *cd47* ^-/-^ kidneys involves cytokine production by Th17 cells. G-CSF (Csf3), GM-CSF (Csf2), GCP2 (Cxcl5) and IL-23 receptor were up-regulated (Table 2). The transcription factors C/EBPβ, Fos and Jun are involved in IL-17 signaling and were correspondingly up-regulated in infected *cd47* ^-/-^ spleens (Table 2). Apoptosis and survival signaling via lymphotoxin-beta receptor was the third most significant in kidney gene expression. TRF2, MAP3K5. MAP3K1 and CCL21b were down regulated in this pathway (Table 2).

## Discussion

In a C57Bl/6J background, *cd47* ^-/-^ mice challenged intravenously with *C*. *albicans* exhibited higher and more rapid lethality. As typically occurs in infected humans and WT mice, kidney colonization limited the survival of infected *cd47* ^-/-^ mice. *C*. *albicans* infection increases neutrophil infiltration into brain and kidneys in WT mice [53,54]. Initial colonization of kidneys was similar in infected WT and *cd47* ^*-/-*^ mice but diverged at day 4. At this time, infected kidneys of *cd47* ^-/-^ mice exhibited excessive neutrophil and macrophage/monocyte infiltration as shown by histopathology and flow cytometry, which is consistent with the observed enlargement of these kidneys. Infected *cd47* ^-/-^ kidneys had more fibrosis, indicating more granulation tissue formation, which may be secondary to the increased inflammation [55]. This was surprising because CD47 was reported to promote PMN recruitment into the peritoneum following *E*. *coli* challenge [20], and integrin-mediated neutrophil migration [19,21]. Neutrophils also accumulated more in *Candida* infected *cd47* ^-/-^ mouse brains. Despite increased neutrophil and macrophage recruitment, the *cd47* ^-/-^ kidneys and brains showed more extensive and invasive *C*. *albicans* colonization. Thus, in contrast to the previous bacterial infection models, where *cd47* ^-/-^ mice displayed a defect in neutrophil recruitment that compromised survival and an endothelium-dependent defect in neutrophil recruitment induced by TNFα [17], we find that CD47 is not necessary for neutrophil recruitment in response to a *C*. *albicans* infection. However, the increased neutrophil and macrophage recruitment in *cd47* ^-/-^ mice is not protective, and CD47 has is no intrinsic role in macrophage phagocytic activity for *C*. *albicans*.

The increased recruitment of innate immune cells at later time points in the infected *cd47* ^-/-^ mice is associated with increased fungal burden in these organs, but in the absence of an identifiable functional phagocytosis or killing defect in the *cd47* ^*-/-*^ innate immune cells, additional CD47-dependent immune defects must enable this increased colonization. An IL-17- and G-CSF-dependent deficit in granulopoiesis in *cd47* ^-/-^ mice was reported in the colitis model [25], but we observed increased neutrophils and macrophages/monocytes in infected *cd47* ^-/-^ kidneys and neutrophils in infected brains. Independent of effects on recruitment, local expansion of granulocytes in infected *cd47* ^-/-^ kidneys may be driven by the increased G-CSF and GM-CSF expression we observed in this organ.

In addition to regulating phagocytic activity by engaging its counter receptor SIRPα [56], CD47 engagement with SIRPα on dendritic cells controls their differentiation and half-life [57,58]. SIRPα is also expressed on normal kidney podocytes, where its basal phosphorylation is decreased following glomerular injury [59]. Thus, the absence of CD47 to engage SIRPα could alter antigen presentation to adaptive immune cells, and signaling between non-immune cells in the kidney. Signaling through SIRPα is generally inhibitory, suggesting that loss of this signal could account for the up-regulation of inflammatory gene expression that we observed in spleens and kidneys of infected *cd47* ^-/-^ mice.

Our data suggests that a defective adaptive immune response also contributes to the increased susceptibility of *cd47* ^-/-^ mice to candidemia. The role of humoral immunity in controlling fungal infections is still understudied but is generally considered to play a minimal role in disseminated candidiasis [60]. Th2 cells produce cytokines that promote antibody production [61], but we did not observe any CD47-dependent alterations in early IgM response up to day 6 PI. Thus, regulation of early humoral immunity is not a major function of CD47 in candidemia.

CD47 is highly expressed on peripheral T lymphocytes and, depending on the context, can be a co-stimulator or inhibitor of T cell activation [21,26,28,62–64]. The present study suggests that CD47 plays a protective role against disseminated candidiasis through regulation of T helper cell differentiation and expression of cytokines that modulate this differentiation. Susceptibility to candidiasis in mice was previously associated with activation of the Th2 subset and IL-10 production [7,65]. Infection of WT mice by a virulent strain of *C*. *albicans* typically results in a 100-fold induction of TNF-α mRNA and several thousand fold induction of IL-6 mRNA [4]. Significant differences in serum cytokine responses to disseminated candidiasis were observed in *cd47* ^-/-^ mice showing severe clinical signs at day 4 PI. Infected *cd47* ^*-/-*^ mice showed significantly higher circulating TNF-α, IL-6, and IL-10 and significantly lower IL-17 levels compared with infected WT. This is consistent with a previous report of diminished serum IL-6 and IL-17A levels in *cd47* ^*-/-*^ mice in a dextran sulfate-induced colitis model [25]. Lower IL-17 is also consistent with a diminished Th17 response reported in *cd47*-null mice exposed to trinitrobenzene sulfonic acid in a colitis model [66]. The same group subsequently showed that treatment with CD47-Fc conferred protection via a Th17-dependent mechanism [67]. Consistent with these data, a recent study by a different group found decreased numbers of Th17 cells in healthy intestinal mucosa of *cd47*-null and SIRPα signaling-deficient mice, and a corresponding defective Th17 response to *Citrobacter* infection [68]. In this context, our data indicates that a defective Th17 response may contribute to the decreased resistance of *cd47*-null mice to *C*. *albicans*.

Despite these changes in serum cytokines, analysis of splenic Th cell subsets at day seven indicates that, as the disease progresses to a chronic stage, *cd47* ^-/-^ mice infected with *C*. *albicans* produce elevated Th1, Th2 and Th17 T cell subsets without altering T reg. This hyper-T cell inflammatory response is not protective and is consistent with the prolonged oxazolone-induced inflammation reported in *cd47* ^-/-^ mice [69]. Interaction between CD47 and BNIP3 induces T cell apoptosis, and the absence of CD47 was reported to prevent termination of a T cell inflammatory response by this mechanism.

Systematic analysis using the NanoString technique provides quantitative and sensitive detection of changes in inflammatory gene expression [47,53]. Cxcl2 (MIP-2 α) and cxcl3 (MIP-2 β) mRNAs were over-expressed 24- and 17-fold, respectively, compared with WT in *cd47* ^-/-^ spleens at day 3. These CXC chemokine family members are chemotactic factors for PMN and other immune effector cells [70].

In vitro studies indicated that *C*. *albicans* induces enhanced levels of MIP-2 [71]. Over-expression of MIP-2 may account for the increased infiltration of inflammatory immune cells into infected *cd47* ^-/-^ kidney and brain, shifting the balance towards a pro-inflammatory milieu. Pathways associated with IL-1, IL-17 signaling and gastrin-mediated inflammatory response were also highly activated in spleens of infected *cd47* ^-/-^ mice. The IL-1 pathway may play a major role in the exaggerated inflammatory response of infected *cd47* ^-/-^ mice. IL-1 is a proinflammatory cytokine produced by activated macrophages, endothelial cells, B cells and fibroblasts, which stimulate a broad spectrum of immune and inflammatory responses. IL-1α & β induce cellular responses through the type I IL-1 receptor (IL-1R1) [72]. Our data show that IL-1α and Il-1R1 mRNA are over-expressed together with important transcription factors in the pathway in infected *cd47* ^-/-^ spleens, leading to more pro-inflammatory reactions. Although IL-1β mRNA is not significantly elevated in infected *cd47* ^-/-^ kidneys, local production of active IL-1β may be elevated because NLRP3, which is a critical element of the inflammasome pathway for proteolytic maturation of IL-1β, is significantly elevated in infected *cd47* ^-/-^ kidneys.

Kidneys of *cd47* ^-/-^ mice infected with *C*. *albicans* at day 3 PI up-regulated csf2 or GM-CSF (8-fold), csf3 or G-CSF (4-fold) and IL-23R (5-fold) and down regulated Ccr2 (7-fold) and Ccl21b (4-fold). The pro-inflammatory environment in the *cd47* ^-/-^ mice may be regulated by changes in Th17 signaling, which was also highly significant in our gene expression analysis. Signaling through this pathway induces IL-6, G-CSF, GM-CSF, and (Cxcl5) GCP2, all of which enhance neutrophil recruitment and granulopoiesis [73], consistent with the significantly higher neutrophil infiltration into infected *cd47* ^-/-^ kidneys and brains in this study. Changes in G-CSF, GM-CSF, GCP2, and the serum cytokine IL-6 in our gene expression analysis strongly correlates with the enlarged severely inflamed kidneys of infected *cd47* ^-/-^ mice.

Collectively, these data demonstrate that *cd47* ^-/-^ mice are more susceptible to disseminated candidiasis. *cd47* ^-/-^ mice exhibit poorly controlled pro-inflammatory responses to *Candida* infection. We propose that CD47 is required in the host to mount a balanced protective immune response against candidiasis. The increased susceptibility of *cd47* ^*-/-*^ mice to *C*. *albicans* contrasts with the resistant phenotype of *thbs1* ^-/-^ mice [34]. This suggests that TSP1 receptors other than CD47 play a dominant role in mediating the effects of TSP1 on a systemic *C*. *albicans* infection. These findings suggest that CD47 could be a useful molecular target for therapeutic intervention to control fungal infections, but the humanized CD47 antibodies that have recently entered clinical trials may adversely affect the control of candidiasis in cancer patients.

## Supporting Information

S1 Data NanoString Data. This Excel file contains the complete raw and processed data supporting Tables 2 and 3. Complete gene expression data normalized using internal controls was determined for each of the infected WT and *cd47*-null spleens and kidneys and is summarized in the “data” worksheet. Genes are listed in order of decreasing significance determined by t-test. Compiled raw nCounter counts for all of the samples analyzed with positive and negative controls are in the “RCC Collection” worksheet. Counts for individual samples are in subsequent worksheets.
(XLS)
